# Inhibition of highly pathogenic PRRSV replication in MARC-145 cells by artificial microRNAs

**DOI:** 10.1186/1743-422X-8-491

**Published:** 2011-11-01

**Authors:** Shuqi Xiao, Qiwei Wang, Jintao Gao, Liangliang Wang, Zuyong He, Delin Mo, Xiaohong Liu, Yaosheng Chen

**Affiliations:** 1 State Key Laboratory of Biocontrol, School of Life Sciences, Sun Yat-sen University, Guangzhou 510006, P. R. China; 2 Pig Improving and Breeding Project Technology Research Exploitation Center of Guangdong, Guangzhou 510006, P. R. China

**Keywords:** Highly pathogenic PRRSV, RNAi, artificial miRNA, Lentivirus

## Abstract

**Background:**

Highly pathogenic porcine reproductive and respiratory syndrome (HP-PRRS) has caused large economic losses in swine industry in recent years. However, current antiviral strategy could not effectively prevent and control this disease. In this research, five artificial microRNAs (amiRNAs) respectively targeted towards ORF5 (amirGP5-243, -370) and ORF6 (amirM-82, -217,-263) were designed and incorporated into a miRNA-based vector that mimics the backbone of murine miR-155 and permits high expression of amiRNAs in a GFP fused form mediated by RNA Pol II promoter CMV.

**Results:**

It was found that amirGP5-370 could effectively inhibit H-PRRSV replication. The amirM-263-M-263, which was a dual pre-amiRNA expression cassette where two amirM-263s were chained, showed stronger virus inhibitory effects than single amirM-263. H-PRRSV replication was inhibited up to 120 hours in the MARC-145 cells which were stably transduced by recombinant lentiviruses (Lenti-amirGP5-370, -amirM-263-M-263). Additionally, efficacious dose of amirGP5-370 and amirM-263 expression did not trigger the innate interferon response.

**Conclusions:**

Our study is the first attempt to suppress H-PRRSV replication in MARC-145 cells through vector-based and lentiviral mediated amiRNAs targeting GP5 or M proteins coding sequences of PRRSV, which indicated that artificial microRNAs and recombinant lentiviruses might be applied to be a new potent anti-PRRSV strategy.

## Background

Porcine reproductive and respiratory syndrome (PRRS) is one of the most significant viral diseases in swine and it threats global swine industries[1]. It has been reported that approximately 560.32 million US dollars lost annually only in the US[2]. The causative of PRRS is porcine reproductive and respiratory syndrome virus (PRRSV), which is an enveloped, single-stranded positive-sense RNA virus and a member of the order *Nidovirales*, family *Arteriviridae*[3]. The PRRSV genome, approximately 15 kb in length, encodes nine partially overlapping open reading frames (ORFs). Among them, ORF5 and ORF6 respectively encodes two PRRSV major envelope structural proteins: a glycosylated major envelope protein GP5 encoded by ORF5 and an unglycosylated membrane M protein encoded by ORF6[4]. Both of these proteins are important to PRRSV infection[5-9], for example, PRRSV M/GP5 complex acts as the ligand to interact macrophage-specific lectin sialoadhesin which is critical for viral infection[10]. In recent years, highly pathogenic porcine reproductive and respiratory syndrome (HP-PRRS) caused by highly pathogenic porcine reproductive and respiratory syndrome virus (H-PRRSV) is endemic in China and has resulted in enormous economic losses in swine-producing areas of the world[11,12]. However, current antiviral strategy could not effectively prevent and control H-PRRSV. Hence, it is imperative to develop a safe and effective antiviral strategy to combat H-PRRSV infection.

RNA interference (RNAi) is a conserved natural mechanism by which homologous small interference RNA (siRNA) duplexes induce potent and sequence-specific posttranscriptional inhibition of gene expression via degradation of complementary messenger RNA (mRNA)[13,14]. Since PRRSV is a RNA virus, its RNA genome is not only the template for viral transcription but also for viral genome replication. Antiviral RNAi might be more potent to be applied to inhibit RNA viruses, such as PRRSV[15,16]. On the other hand, due to PRRSV infection in pigs is time persistent[17], there is a need to use stable antiviral RNAi therapy to durably protect pigs to combat PRRSV infection. Lentiviral vectors allow expressing exogenous DNA to induce stable and long-term gene silence in dividing and non-dividing cells[18,19]. RNAi against viral infection based on lentiviral delivery has been intensively investigated and evaluated for potential therapeutic applications in HIV-1, Hepatitis viruses (Hepatitis B, HBV and Hepatitis C, HCV), Human papilloma virus, Coxsackie virus, Encephalitogenic flaviviruses, Prion disease, etc (reviewed in [20]). Recently, artificial microRNAs (amiRNAs) have been shown to be more effective than conventional short hairpin RNA (shRNA) as an antiviral strategy[20-25].

In this research, two recombinant plasmids expressing amiRNAs, which are designed to be perfectly homologous to PRRSV GP5 and M proteins coding sequences respectively, were found to be capable of inhibiting H-PRRSV replication without inducing innate interferon response. Additionally, it was found that H-PRRSV replication could be effectively inhibited in MARC-145 cells up to 120 h at least by stably transduced with recombinant lentiviruses expressing verified amiRNAs.

## Results

### AmiRNAs design and target sites selection using luciferase assay

**Figure 1:**
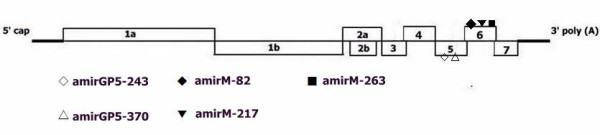
**Genomic structure of PRRSV and target positions of artificial microRNAs (amiRNAs)**.

**Figure 2:**
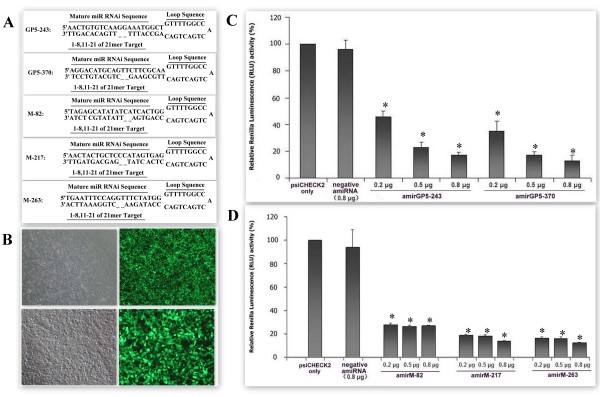
**Designed amiRNAs and target sites selection using luciferase assay**. Five amiRNAs were designed via online tools of Invitrogen Company (A). Transfection efficiency assay via GFP monitoring after co-transfected with plasmids expressing amiRNAs that fused to GFP coding gene and psiCHECK2-GP5 or -M. Images were taken with an inverted fluorescence microscope in bright (left) or fluorescence (right) light of the same field. Magnification, 40× (upper) or 100 × (bottom) (B). Inhibition of *Renilla *luciferase expression by amiRNAs that targeting PRRSV GP5 or M protein coding sequences were measured by duel luciferase assay at 48 hours post-cotransfection. The *Renilla *luciferase data has been normalized to firefly luciferase data. Normalized *Renilla *luciferase expression in the psiCHECK2 only transfection was set at 100%. Data are mean ± SD, n = 3~4, *p < 0.05 vs. psiCHECK alone transfected cells group (C, D).

Artificial microRNAs (amiRNAs), which were designed using online tools (Invitrogen), target PRRSV GP5 and M proteins coding sequences, as shown in Figure 1 and 2A. Then recombinant plasmids expressing amiRNAs were constructed and co-transfected with duel-luciferase report plasmids psiCHECK2-GP5 or psiCHECK2-M into 293FT cells using lipofectamine 2000 (Invitrogen), as shown in Figure 2B. In the luciferase assay, *Renilla *luciferase signals were normalized to the firefly luciferase signals in order to correct the differences of transfection efficiencies. Relative luciferase expression in the presence of psiCHECK2 alone was set at 100%. We observed that inhibitory effects of all the amiRNAs were more than 70%. The relative renilla luminescence (RLU) activities in amiRNAs transfected cells were significantly lower than that in the cells transfected with psiCHECK2 alone (p < 0.05). Negative amiRNA transfected cells did not show significantly reduction in RLU activity compare to psiCHECK2 alone transfected cells (Figure 2C and 2D). Moreover, expect for amir-82, the inhibitory of amiRNAs were dose-dependent (Figure 2C and 2D).

### Construction of plasmids and titering lentiviral stock

Six amiRNAs expressing plasmids (pcDNA™6.2-GW/EmGFP-amirGP5-243; -amirGP5-370; -amirM-82; -amirM-217; -amirM-263; -amirM-263-M-263), two luciferase report plasmids psiCHECK2-GP5 and psiCHECK2-M, and three recombinant lentiviral expression plasmids (pLenti6/V5-GW/EmGFP-amirGP5-370; -amirM-263-M-263; -negative amiRNA) were constructed. All the nucleotides sequences inserted into vectors were confirmed by sequencing. The titers of the three recombinant lentiviruses were 3.35 × 10^6 ^TU/ml (Lenti-amirGP5-370), 3.33 × 10^6 ^TU/ml (Lenti-amirM-263-M-263), 1.81 × 10^6 ^TU/ml (Lenti-negative amiRNA).

### Inhibition of cytopathic effect (CPE) induced by H-PRRSV in MARC-145

**Figure 3:**
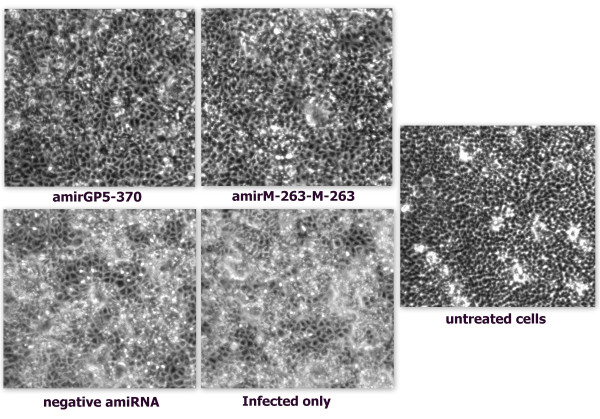
**CPE analysis of H-PRRSV on MARC-145 cells transfected with pcDNATM6.2-GW/EmGFP -amirGP5-370, -amirM-263-M-263 or -negative amiRNA**. Cells were challenged with H-PRRSV at 0.01 MOI and incubated for 60 hours, Magnification 100×.

To determine whether amiRNAs could inhibit CPE induced by H-PRRSV and protect MARC-145 cells, MARC-145 cells were transfected with plasmids expressing amiRNAs that target PRRSV GP5 and M protein coding sequence. In parallel, a nonspecific amiRNA that does not target any known vertebrate genes was transfected. Transfection efficiency was determined by GFP fluorescence intensity of transfected cells on an inverted fluorescence microscope. At six hours after transfection, MARC-145 cells were infected with H-PRRSV. Sixty-hour post-infection, we found that cells pre-transfected with pcDNA™6.2-GW/EmGFP-amirGP5-370 or pcDNA™6.2-GW/EmGFP-amirM-263-M-263 showed less CPE compared to others. MARC-145 cells treated with pcDNA™6.2-GW/EmGFP-neagtive-amiRNA demonstrated the same typical PRRSV-induced CPE as cells that infected with H-PRRSV alone (positive control), which became reticulated and detached from the monolayer, as shown in Figure 3.

### **AmiRNAs specifically **silence **PRRSV genes in H-PRRSV infected MARC-145 cells**

**Figure 4:**
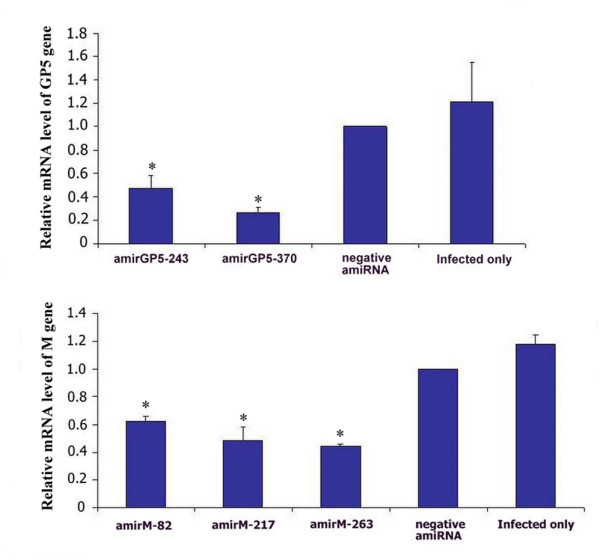
**Inhibition on H-PRRSV GP5 and M expression by amiRNAs were observed by qPCR**. The mRNA of β-actin was served as an internal reference. GP5 and M protein coding genes expression normalized to β-actin gene in negative amiRNA transfected cells group was set at 100%. Data are mean ± SD, n = 3, *p < 0.05 vs. negative amiRNAs alone transfected cells group.

**Figure 5:**
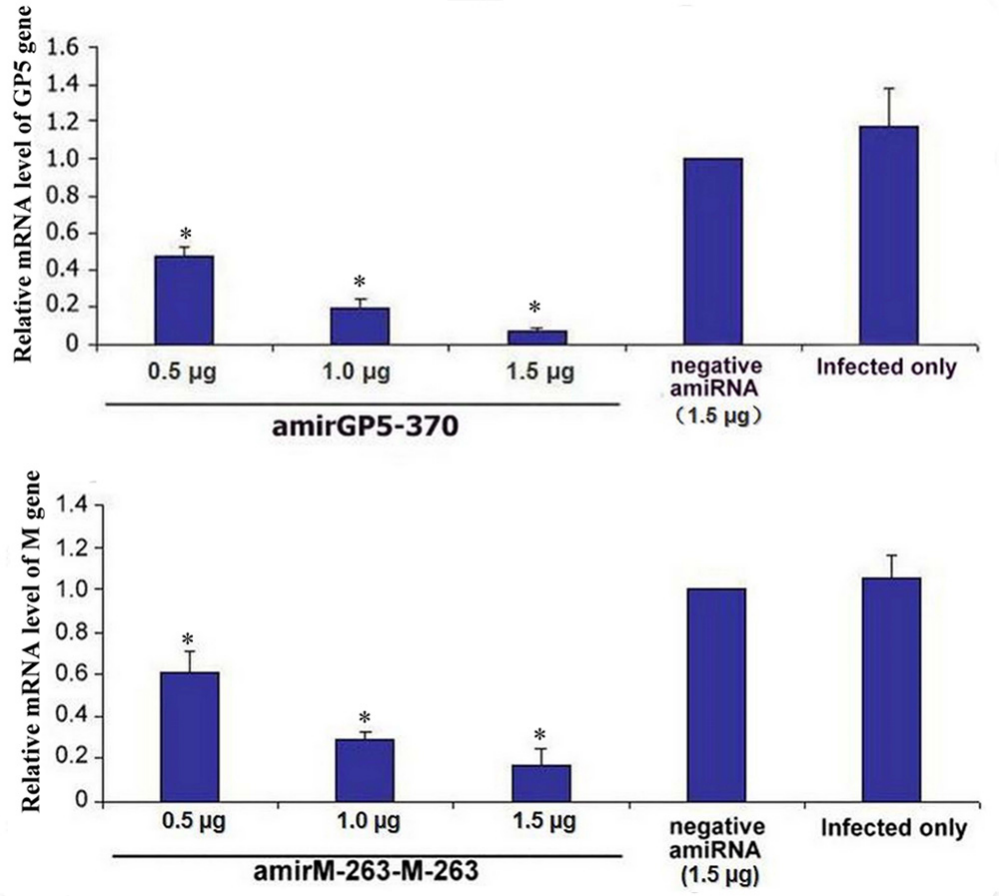
**Dose-dependent inhibition on specific H-PRRSV gene expression by amirGP5-370 and amirM-263-M-263 was observed by qPCR**. The mRNA of β-actin was served as an internal reference. GP5 and M protein coding genes expression normalized to β-actin gene in negative amiRNA transfected cells group was set at 100%. Data are mean ± SD, n = 3~4, *p < 0.05 vs. negative amiRNAs alone transfected cells group.

To determine whether the viral suppression was due to specific viral gene expression inhibition, the cells were infected with H-PRRSV at six hours post-transfected with amiRNAs expressing plasmids. At sixty-hour post-infection, the cells were harvested to detect H-PRRSV GP5 or M protein coding genes and β-actin gene in each experimental group by real-time PCR. β-actin gene was served as an internal reference. AmirGP5-370 showed highest inhibition of expression of its target gene (GP5 protein coding gene) in H-PRRSV infected MARC-145 cells (p < 0.05) (Figure 4). AmirM-263-M-263, which was a duel pre-amiRNA in one expression cassette chained by two amirM-263s, was found to have higher inhibitory effect than single amirM-263 (Figure 4 and Figure 5). It was also found that expression of H-PRRSV GP5 and M protein coding genes were down regulated in dose-dependent by amirGP5-370 and amirM-263-M-263, respectively, compared to the negative amiRNA treated and H-PRRSV infected alone groups (Figure 5).

**Figure 6:**
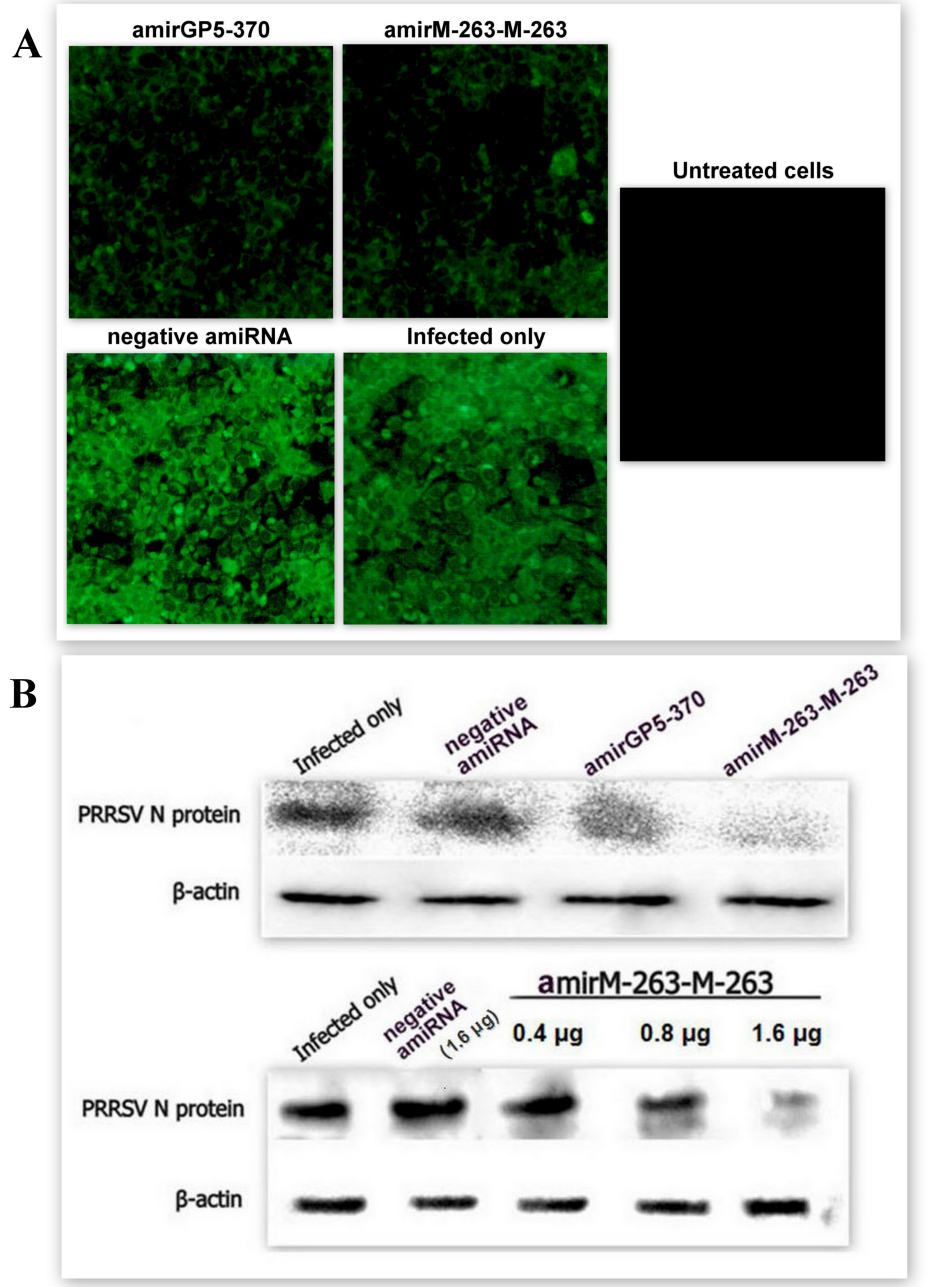
**AmiRNAs expression plasmids inhibit specific H-PRRSV protein expression in MARC-145 cells**. Cells seeded in 96-well (for indirect fluorescence assay, IFA) or 12-well (for Western blotting, WB) plates were transfected amiRNAs expression plasmids, and infected with H-PRRSV at 0.01 MOI at six-hour post-infection. At sixty hours after infection, PRRSV was detected by IFA and WB with PRRSV positive serum isolated from SPF pigs. Pictures were taken with an inverted fluorescence microscope. Magnification, 100×(A). Whole cell extracts of experimental groups were prepared for WB and β-actin protein was served as an internal reference (B). The data here was one represent of three experiments.

To investigate viral proteins expression, PRRSV were detected by IFA and WB with PRRSV positive serum isolated from SPF pigs. As shown in Figure 6A, compared with infected alone group and negative amiRNA treated group, amirGP5-370 and amirM-263-M-263 greatly reduced frequency of fluorescence stained MARC-145 cells in IFA assay. It was observed that N protein amount in amirGP5-370 or amirM-263-M-263 treated groups are greatly reduced than infected alone and negative amiRNA treated groups (Figure 6B). Moreover, the inhibitory effect of amirM-263-M-263 was still dose dependent(Figure 6B).

### Interferon response is not triggered by efficacious amiRNAs

**Figure 7:**
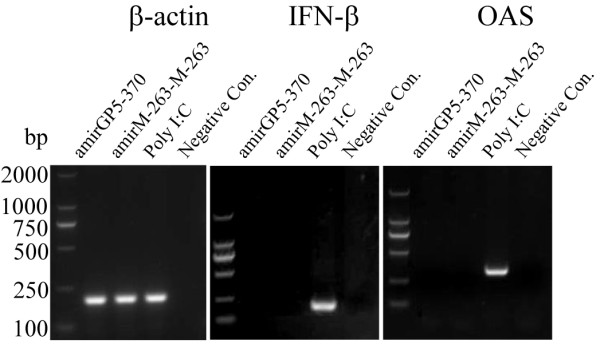
**Artificial microRNAs do not trigger IFN activation**.

Double stranded RNA (dsRNA) [26] may induce innate immune response, and activation of the type I interferon (IFN) pathway by small interfering RNA (siRNA) is a major contributor to the off-target effects of RNA interference in mammalian cells[27]. To detect possible induction of innate immune response by amiRNAs, RT-PCR analysis of IFN-β and 2,5-oligoadenylate synthetase (**OAS**) were carried out in the experiment groups that were transfected efficacious dose amiRNAs expressing plasmids. It was observed that amplifications of IFN-β and OAS were absence in the ethidium bromide stained agarose gels (Figure 7). Meanwhile cells treated with poly I:C was set as positive control, in which IFN-β and OAS genes can be detected (Figure 7).

### Duration of inhibitory effects on H-PRRSV in MARC-145 cells that stably transduced with Lenti-amiRNAs

**Figure 8:**
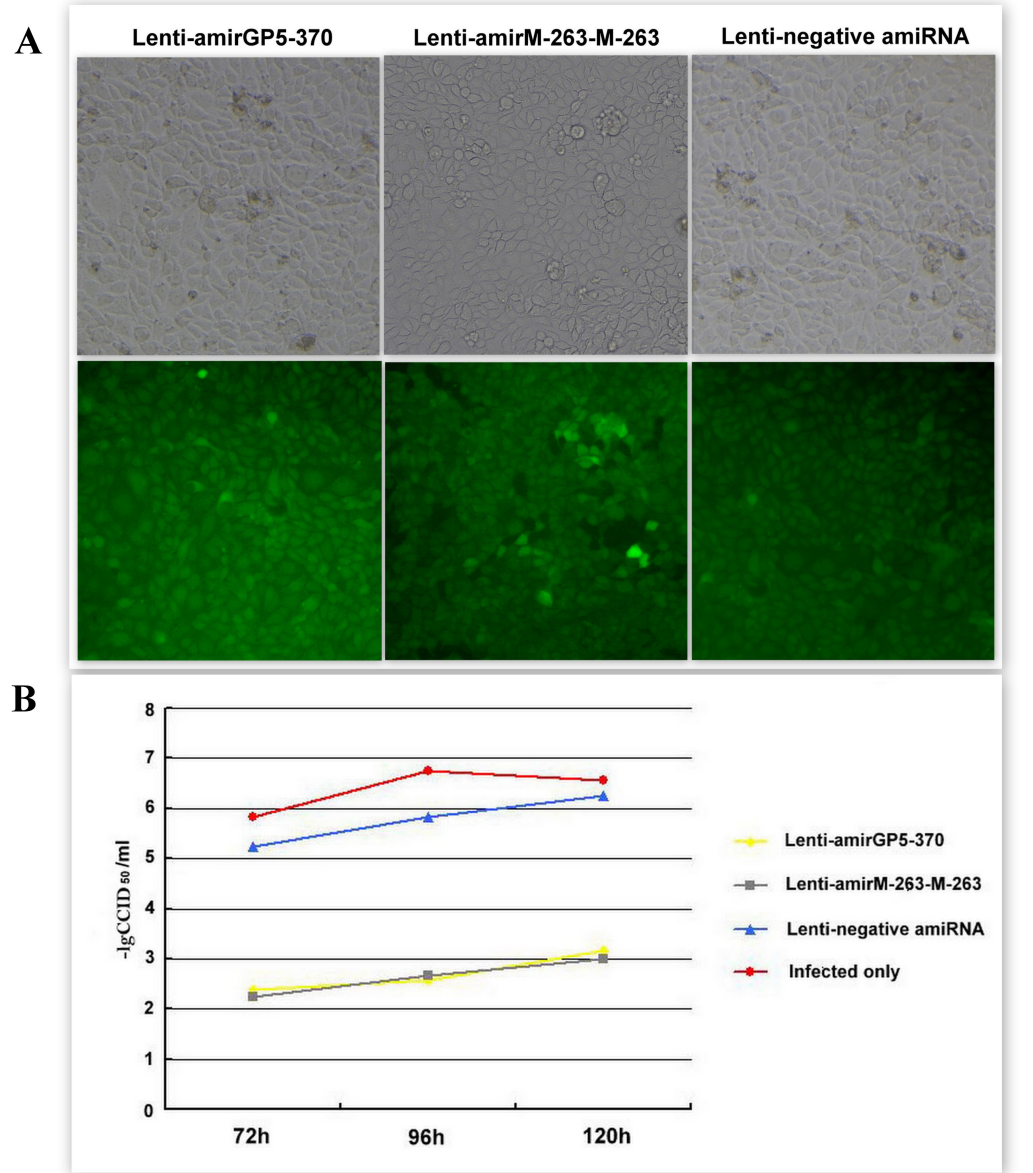
**Duration of inhibitory effects on H-PRRSV in MARC-145 cells that stably transduced with Lenti-amiRNAs**. Stably transduced MARC-145 cells were generated by recombinant lentiviruses via Blasticidin (8 μg/ml) selection. Images were taken with an inverted fluorescence microscope in bright (upper) or fluorescence (bottom) light of the same field, Magnification 200× (A). MARC-145 cells that were stably transduced with amirGP5-370, amirM-263-M-263 and negative amiRNA were infected with H-PRRSV at 5.0MOI. At 72, 96 and 120 hours post-infection, cell cultures were harvested, respectively, and virus titers were assayed by CCID_50 _(B).

To analyze the persistent inhibition of H-PRRSV by amiRNAs, stably transduced MARC-145 cells with Lenti-amirGP5-370, Lenti-amirM-263-M-263 or Lenti-negative amiRNA were selected by using 8 μg/ml Blasticidin. Blasticidin-resistant colonies were picked and expanded. MARC-145 cells that stably express amirGP5-370, amirM-263-M-263 or negative amiRNA were infected with H-PRRSV at 5.0MOI. Cell cultures were harvested at 72, 96 and 120 h post-infection and then were assayed for CCID_50_. We found that H-PRRSV titers were at least approximately 1000-fold lower in MARC-145 cells expressing amirGP5-370 or amirM-263-M-263 compared to those infected with H-PRRSV alone or expressing negative amiRNA (Figure 8).

## Discussion

Vaccination is the principal means used to control and treat PRRSV infection. An array of PRRS vaccines have been developed, but these all could not provide sustainable disease control because they suffer both from the immune evasion strategies of the virus and the antigenic heterogeneity of field strains[28]. Hence, it is imperative to develop a safe and effective antiviral strategy to combat PRRSV infection. RNAi is a process of gene silencing which can be induced by intracellular expression of short hairpin RNA (shRNA) or artificial microRNA (amiRNA) delived by vectors or viruses. Previous researches have been demonstrated that PRRSV replication could be inhibited by RNAi induced by recombinant plasmids or adenoviruses expressing shRNAs[29-33]. Chen *et al*. (2006) demonstrated that adenovirus-mediated FMDV-specific shRNA could significantly reduce the susceptibility of swine to FMDV infection[34]. Carmona *et al*. (2006) demonstrated that anti-HBx shRNA could effectively inhibited HBV replication[35].

However, shRNAs are not the optimal substrate for RNAi process in that shRNAs will not be processed by Drosha that could create cleavage sites for further cleave by Dicer which is critical for efficient RNAi effect[36]. Therefore, it is required many candidate shRNA sequences to identify the effective ones. On the other hand, shRNA expressing vectors always use Polymerase III promoters, which not only limits tissue-specific expression of shRNA but also has shown to have higher possibilities to induce cellular toxicities due to over expression of shRNA driven by Polymerase III promoters might interfere with endogenous microRNA biogenesis[37].

Recently, pre-artificial microRNAs (pre-amiRNA) driven by Polymerase II promoters that via naturally existing endogenous microRNA pathway to become mature amiRNA have shown to be more potent antiviral RNAi inducers but with less dangerous of toxicities compared to conventional shRNAs[20-25]. RNA polymerase II is tightly regulated and it can drive tissue-specific amiRNA expression. In addition, endogenous microRNA pathway saturation caused by overexpression of exogenous shRNAs will be avoided by utilizing RNA polymerase II driven amiRNA expression constructs[38-41]. However, this has not been applied in the anti-PRRSV RNAi strategy.

In this research, five amiRNAs (amirGP5-243, amirGP5-370, amirM-82, amirM-217 and amirM-263) targeting PRRSV GP5 or M proteins coding sequences were designed via online tool. Then recombinant plasmids expressing theses amiRNAs were constructed. In the duel luciferase signal assay experiment, we observed that all amiRNAs could inhibit *Renilla *luciferase signal at least 70% via cleavage their target sequences. Except for amirM-82, inhibitory effects of all the other amiRNAs were found to be in dose dependent (Figure 2C and 2D). Our results indicated that amiRNAs-mediated RNAi might be a potent method to induce gene silence.

To investigate whether amiRNAs could inhibit accumulation of viral mRNAs or proteins of target genes, we carried out CPE analysis, real-time PCR, IFA and Western blotting assays. Among five amiRNAs tested in this study, amirGP5-370 showed the highest inhibitory effect on H-PRRSV replication and ORF5 gene expression in MARC-145 cells infected with H-PRRSV (Figure 4). Other four amiRNAs that were verified to be able to cleave target gene in duel luciferase assays failed to effectively knock down target viral genes in MARC-145 cells with H-PRRSV infection. It may be because of limited transfection efficiency and rapid replication of H-PRRSV. Efficiency of transfection of plasmids into MARC-145 cells using Lipofectamine 2000 in this study, which are measured by GFP fluorescence intensity, is approximately more than 50% (data not shown). Although this transfection efficiency is slightly higher than previous study using MARC-145 cells[30], it is still low and will underestimate the inhibition effects due to viral replication in those MARC-145 cells that failed to be transfected with amiRNAs. This case became more obvious when cells infected with H-PRRSV that will induce highly pathogenic lesions in cells. To combat rapid replication of H-PRRSV, increased inhibitory effects on target sequence are needed.

Chaining amiRNAs in one expression construct is permitted for the plasmids we used in this research. It also has been shown that increased gene knock-down effects could be induced by repeating same amiRNA in one expression construct. Among the other four amiRNAs, which showed less effective inhibition on H-PRRSV replication, amirM-263 showed greatest inhibitory effects. In this case, we chained two amirM-263s to construct a duel pre-amiRNAs construct amirM-263-M-263. It was observed that amirM-263-M-263 showed much higher inhibitory effects on viral gene transcription compared to amirM-263, and could inhibit H-PRRSV replication in MARC-145 cells (Figure 5 and 6).

Plasmids or adenoviruses delivery strategy is limited by its transient nature, as a result, RNAi effects were reduced over time post-infected with PRRSV and PRRSV could replicate afterwards. Also, PRRSV might develop certain mechanism to escape RNAi effect[32], but it is difficult to investigate possible virus escape in the condition of transient antiviral RNAi. Moreover, it has been shown that PRRSV infection can persist in pigs after preliminary infection[17]. Taken together, antiviral RNAi against PRRSV infection should be a relatively stable therapy that can durably protect pigs. Lentiviral vector is a powerful DNA delivery tool that allows expressing exogenous DNA to induce stable and long-term gene silence in dividing and non-dividing cells[18,19]. To date, lentiviral mediated RNAi have been intensively investigated for combat viral infection (reviewed in[20]), but has not been reported to be used as an anti-PRRSV strategy. In this research, one of our aims is to investigate whether lentiviral mediated antiviral RNAi could persistently inhibit H-PRRSV replication in MARC-145 cells. Three recombinant lentiviruses expressing amirGP5-370, amirM-263-M-263 and negative amiRNA, respectively, were produced in 293FT cells. The results showed that H-PRRSV replication would be suppressed in MARC-145 cells stably expressed amirGP5-370 or amirM-263-M-263 up to 120 hours post infection (Figure 8).

Small interfering RNA (siRNA) may induce type I interferon (IFN-I) activation, which will result in off-target effects of RNAi in mammalian cells[27]. However, in our research, efficacious dose of amirGP5 and amirM-263-M-263 will not trigger interferon response (Figure 7).

## Conclusions

We designed five artificial amiRNAs respectively targeting PRRSV GP5 (amirGP5-243, -370) or M (amirM-82, -217,-263) proteins coding sequences and constructed recombinant plasmids expressing theses amiRNA. The results showed that amirGP5-370 and amirM-263-M-263 could effectively suppress H-PRRSV replication without inducing innate interferon response. And H-PRRSV replication could be effectively inhibited in MARC-145 cells up to 120 h at least by stably transduced with recombinant lentiviruses expressing verified amiRNAs. To our knowledge, the study presented here is the first attempt to suppress H-PRRSV replication in MARC-145 cells through vector-based and lentiviral mediated amiRNAs targeting GP5 or M proteins coding sequences of PRRSV. The study indicated that artificial microRNAs and recombinant lentiviruses might be applied to be a new potent anti-PRRSV strategy.

## Methods

### Design of amiRNAs and construction of plasmids expressing amiRNAs

Five precursor miRNAs (pre-miRNA) sequences respectively targeting on PRRSV GP5 and M protein coding genes were designed through an internet application system (Invitrogen). Double-stranded oligonucleotide encoding pre-miRNA sequence were annealed and inserted into pcDNA™6.2-GW/EmGFP-miR expression vector (Invitrogen) containing cytomegalovirus (CMV) promoter and herpes simplex virus thymidine kinase polyadenylation signal. All recombinant plasmids have been sequenced to confirm the sequences inserted.

### Cell cultures, transfection and virus infection

African green monkey kidney cell line MARC-145 were applied to grow PRRSV and to determine virus titers. Human embryo kidney cells (293FT) were applied to produce lentivirus. MARC-145 cells and 293FT cells were both cultured in Dulbecco modified Eagle medium (DMEM) supplemented with 10% Fetal Bovine Serum (FBS), 2 mM L-glutamine, 0.1 mM MEM Non-Essential Amino Acids, 1% penicillin/streptomycin, and 1 mM MEM Sodium Pyruvate. Cells were typsinized and seeded in 96-well, 12-well or 6-well plate nearly 24 hours before tansfection. For transfection, lipofectamine 2000 (Invitrogen) was used according to manufacturer's instruction. Six hours post-transfection, MARC-145 cells were infected with Highly pathogenic PRRSV GD isolate (kindly provided by Dr. Guihong Zhang in South China Agricultural University, China) at a multiplicity of infection of 0.01 as described in previous study[30].

### Dual luciferase reporter assays

Luciferase reporter assays were performed using the psiCHECK2-GP5 and psiCHECK2-M. 293FT cells were grown to approximately 80% confluence in 24-well plates and cotransfected with psiCHECK2-GP5, psiCHECK2-M or psiCHECK2 empty vector plus 0.2 μg, 0.5 μg or 0.8 μg pcDNA™6.2-GW/EmGFP-miR. Cells were incubated with transfection reagent lipofectamine 2000/DNA complex for 6 hours and then refreshed with fresh DMEM containing 1% FBS for 48 hours. Firefly and *Renilla *luciferase activities were evaluated using the Dual-Luciferase Reporter Assay system (Promega), and *Renilla *luciferase activity was normalized to firefly luciferase activity.

### Virus titration

MARC-145 cells were typsinized and seeded in 96-well plate 24 hours before virus infection. Virus supernatants were 10-fold serially diluted and added 100 μl to each well in eight repeated. Six days after infection, the 50% cell culture infection dose (CCID_50_) was calculated by the Reed-Muench method.

### Analysis of PRRSV GP5 and M protein coding genes by real-time PCR

Total RNA was isolated from MARC-145 cells at 60 h after H-PRRSV infection as described above using RNApreppure total cell RNA isolation kit (Tiangen, Beijing, China). Genomic DNA was removed using DNase I (NEB). 2 μg RNA was reverse-transcribed into first strand cDNA with M-MLV transcriptase (Promega) and oligo d(T)_18 _primers (TaKaRa). 1 μl cDNA was submitted to real-time PCR analysis using specific primers for β-actin, PRRSV GP5 and M protein coding genes. β-actin-F:5'TGACTGACTACCTCATGAAGATCC3';β-actin-R:5'TCTCCTTAATGTCACGCACGATT3'[30], GP5-F:5'ACTCACCACCAGCCATTTC3';GP5-R: 5' CAGTTCTTCGCAAGCCTAA 3', M-F: 5' CACCTCCAGATGCCGTTTG 3'; M-R: 5' ATGCGTGGTTATCATTTGCC 3', and SYBR^® ^Premix Ex Taq™ (TaKaRa). Real-time PCR was performed in a LightCycler^® ^480 Real-Time PCR System and analyzed with LightCycler^® ^480software (Roche). Amplification was carried out in a 10 μl reaction mixture containing 5 μl SYBR^® ^*Premix Ex Taq*™ (TaKaRa) 2×, 0.2 μM concentration of each primer, 1 μl cDNA. The reaction procedure was 95°C 10s, followed by 40 cycles at 95°C for 5s and 60°C for 40s. β-actin gene was served as an internal reference. To confirm specific amplification, melting curve analysis was performed.

### Indirect immunofluorescence

MARC-145 cells seeded in 96-well plate were transfected with pcDNA™6.2-GW/EmGFP-amiR-GP5-370, pcDNA™6.2-GW/EmGFP-amiR-M-263-M-263 or pcDNA™6.2-GW/EmGFP-amiR-negative, and then were infected with H-PRRSV. At 60 hours after virus infection, MARC-145 cells were fixed with pre-cooled methanol for 20 minutes on ice. Following three washes by phosphate-buffer saline (PBS, pH7.4), the fixed MAR-145 cells were incubated with PRRSV positive serum for 2 hours at 37°C. Unbound antibodies were washed three times with TBST. Then, FITC labeled goat anti-pig IgG antibody (KPL) was added and incubated for 1 hour at 37°C. After 3 washes by TBST, fluorescence was analyzed using a fluorescence microscopy (Nikon).

### Western blotting analysis

Western blotting was processed as described previously[42]. The PVDF membrane was probed with a 1:100 dilution of PRRSV positive serum. To normalize protein loading, the PVDF membrane was simultaneously incubated with mouse β-actin monoclonal antibody (BioVision, CA, USA) at a dilution of 1: 4,000. The horseradish peroxidase-conjugated goat anti-pig IgG at a dilution of 1:1,000 and horseradish peroxidase-conjugated goat anti-mouse IgG at a dilution of 1:5,000 was used as secondary antibodies. The protein bands were visualized using supersignal west pico chemiluminescence substrate (Pierce, IL, USA) and Image Quant RT ECL detector (GE).

### RT-PCR for interferon assay

293FT cells were respectively transfected with 1 μg amiRNA construct in 24-well plate (pcDNA™6.2-GW/EmGFP-amiR-GP5-370 or pcDNA™6.2-GW/EmGFP-amiR-M-263-M-263) using Lipofectamine 2000 (Invitrogen). The 2 μg poly I:C (Sigma) that was transfected into 293FT cells in 24-well plate served as a positive control for innate response induction as described previously[25] and native 293FT cells served as a negative control. Total RNA was isolated from 293FT cells 24 h post-treated using RNApreppure total cell RNA isolation kit (Tiangen, Beijing, China). Genomic DNA was removed using DNase I (NEB). RNA (2 μg) was reverse-transcribed into first strand cDNA with M-MLV transcriptase (Promega) and random hexamer primers (TaKaRa).

PCR amplification was performed on 1 μl RT product with interferon-β(IFN-β), 2',5'-oligoadenylate synthetase (OAS) and β-actin(endogenous control) specific primers. IFN-β-F: 5' GATTCATCTAGCACTGGCTGG 3'; IFN-β-R: 5' CTTCAGGTAATGCAGAATCC 3'(186 bp)[43], OAS-F: 5' AGTGCATCTTGGGGGAAAG 3'; OAS-R: 5' CATTACCCTCCCATCAGGTGC 3' (302 bp) and β-actin-F: 5' GACTACCTCATGAAGATCCTCAC 3'; β-actin-R: 5' ATTGCCAATGGTGATGACCTG 3' (197 bp) [43].

### Production of recombinant adenoviruses

The pDONR™ 211 vector was used as intermediate to transfer the pre-amiRNA expression cassette constructed before into the lentiviral expression plasmid (pLenti6/V5-DEST) using Gateway technology (Invitrogen) to generate pLenti6/V5-GW/EmGFP-miR. The pLenti6/V5-GW/EmGFP-miR then was co-transfected with packaging vectors (Invitrogen) into 293FT cells using lipofectamine 2000 (Invitrogen). The cell supernatants were collected at 72 h post-transfection and used as a virus stock. All lentiviruses expressed enhanced green fluorescence protein (GFP), allowing for titering and measuring their infection efficiency in transfected cells. The viral titers were determined by counting GFP-positive cells after transduced in the presence of polybrene (6 μg).

### Statistics

Statistical significance was determined by student's t-test. A P-value<0.05 was considered statistical significant. N values represent the number independent experiments.

## List of abbreviations

HP-PRRS: Highly pathogenic porcine reproductive and respiratory syndrome; amiRNAs: artificial microRNAs; PRRSV: porcine reproductive and respiratory syndrome virus; H-PRRSV: highly pathogenic porcine reproductive and respiratory syndrome virus; RNAi: RNA interference; siRNA: small interference RNA; shRNA: short hairpin RNA; CPE: cytopathic effect; pre-amiRNA: pre-artificial microRNAs; CCID50: 50% cell culture infection dose; GFP: green fluorescence protein.

## Competing interests

The authors declare that they have no competing interests.

## Authors' contributions

SX and QW conceived and designed the study. SX and QW performed the experiments, analyzed data, and wrote the manuscript. JG, LW, ZH and DM coordinated the study. YC and XL contributed to the interpretation of the results and took part to the critical revision of the manuscript. All authors read and approved the final manuscript.
